# The role of the preoperative HALP score in predicting pathological T and N categories in colon cancer: a single-center retrospective study

**DOI:** 10.3389/fonc.2026.1866931

**Published:** 2026-08-05

**Authors:** Çağan Aykurt, Ali Bal, Abdülkadir Ünsal, Serdar Çoban, Adem Ozcan, Gökhan Akkurt

**Affiliations:** Surgical Oncology Clinic, Ankara Bilkent City Hospital, Ankara, Türkiye

**Keywords:** colon cancer, HALP score, immunonutritional biomarker, pathological N category, pathological T category

## Abstract

**Objective:**

The HALP score is an easily accessible immunonutritional biomarker that may reflect systemic inflammation and nutritional status. This study aimed to evaluate the association of the preoperative HALP score with pathological T (pT) and N (pN) categories in patients with colon cancer. In addition, the performance of the HALP score in distinguishing between early (pT1–2) and advanced (pT3–4) T categories was investigated.

**Methods:**

This single-center retrospective study included 100 patients who underwent elective surgery for nonmetastatic colon cancer between December 2020 and December 2025. Patients who received neoadjuvant treatment, had M1 disease, or underwent emergency surgery because of bleeding or obstruction were excluded. The HALP score was calculated using hemoglobin, albumin, lymphocyte, and platelet values obtained from peripheral venous blood within one week before surgery. Discriminative performance was evaluated using ROC analysis, and cut-off values were determined using the Youden index.

**Results:**

The patients were evaluated in three comparison groups: in Group 1, patients with pN0 disease were compared with those with pN+ disease; in Group 2, patients with pT1–2/pN0 disease were compared with all other patients; and in Group 3, patients with pT1–2 disease were compared with those with pT3–4 disease, irrespective of pN status. The discriminative performance of the HALP score was not significant in Group 1 (AUC = 0.53; 95% CI: 0.42–0.65; p = 0.568), whereas the AUC was 0.77 in Group 2 (95% CI: 0.64–0.89; p < 0.001) and 0.81 in Group 3 (95% CI: 0.71–0.92; p < 0.001). At the cut-off value of 55.37 determined for Groups 2 and 3, sensitivity was 55.0% and 60.0%, respectively, while specificity was 91.2% and 96.0%, respectively. These results demonstrated that Group 3 had statistically better diagnostic performance than Group 2.

**Conclusion:**

In colon cancer, the HALP score was able to distinguish between early (pT1–2) and advanced (pT3–4) T categories, irrespective of pN status. Our findings suggest that the HALP score may serve as an adjunctive biomarker that could contribute to preoperative risk assessment in patients with colon cancer. The study findings should be validated in larger, prospective, multicenter cohorts.

## Introduction

Colon cancer is one of the most common malignancies worldwide, and recurrence and overall survival outcomes vary markedly according to tumor stage after surgical treatment. Therefore, there is growing interest in easily applicable and low-cost biomarkers that can predict the pathological stage of colon cancer in the preoperative period. Systemic inflammation and nutritional status have been reported to be associated with tumor progression and may have prognostic value in colon and rectal cancers (1, 2). In particular, composite scores that simultaneously reflect the immune response, inflammation, and nutritional reserve are considered to represent tumor biology more comprehensively than individual inflammatory parameters. The HALP score, calculated using hemoglobin, albumin, lymphocyte, and platelet values, is a notable immunonutritional biomarker in this regard (3). Each component of the HALP score reflects a distinct pathophysiological process associated with tumor biology. Hemoglobin levels reflect cancer-related anemia and tissue hypoxia; albumin levels reflect nutritional reserve and systemic inflammation; lymphocyte count reflects the adequacy of the antitumor immune response; and platelet count reflects tumor-related inflammation, angiogenesis, and the potential for metastatic spread (4). Therefore, a low HALP score is considered to be associated with more aggressive tumor biology, whereas a high HALP score has been associated with more favorable oncological outcomes.

In the literature, the HALP score has frequently been evaluated in relation to overall survival, TNM stage, and disease prognosis. However, whether the association between the HALP score and pathological T (pT) category is independent of nodal status, tumor stage, and demographic variables has not been sufficiently investigated. In this study, the performance of the HALP score in distinguishing pT categories independently of nodal status, tumor stage, and demographic variables was examined using ROC analysis. The HALP score was evaluated as a continuous variable, a multivariable model was constructed for pT category, and a separate analysis was performed for pathological N (pN) categories. The data-derived cut-off value was subjected to internal validation using the bootstrap method.

## Materials and methods

### Study design and patient population

This retrospective cohort study evaluated data from patients who underwent elective surgery for colon cancer at a tertiary care center between December 2020 and December 2025. During the study period, 150 patients were assessed for eligibility; after the inclusion and exclusion criteria were applied, 100 patients were included in the final analysis.

Patients who underwent elective surgery for colon malignancy, had the laboratory measurements required to calculate the HALP score within one week before surgery, had not received neoadjuvant treatment, and had no clinically or radiologically detected distant metastases were included. A total of 50 patients who had received neoadjuvant treatment, underwent emergency surgery because of bleeding or obstruction, had M1 disease, or had a history of hematological disease, chronic inflammatory disease, or another malignancy were excluded. There were no missing data for age, sex, HALP score, pathological T category, or pathological N status in the final analytical dataset.

The HALP score in colon cancer was not evaluated separately according to the segmental location of the tumor; tumors located in different colonic segments were analyzed within a single colon cancer group. To reduce the potential confounding effect of neoadjuvant treatment on the components of the HALP score and to create a more homogeneous patient population, patients who had received neoadjuvant treatment were excluded.

Pathological T and N categories and stage grouping were determined according to the 8th edition of the AJCC TNM classification. Distant organ metastases may affect the HALP score by altering hematological and nutritional parameters through their systemic effects. In addition, some patients with metastatic disease may be referred for neoadjuvant treatment before surgery, which may lead to changes in the components of the HALP score. To reduce these confounding effects and create a more homogeneous patient population, patients with M1 disease were excluded. Therefore, all patients included in the analysis had M0 disease. According to stage grouping, 20/100 patients (20.0%) were classified as stage I, 23/100 (23.0%) as stage II, and 57/100 (57.0%) as stage III, and there were no patients with stage IV disease.

The HALP score was calculated using the following formula:

Hemoglobin (g/L) × Albumin (g/L) × Lymphocyte count (×10^9^/L)/Platelet count (×10^9^/L).

Hemoglobin, albumin, lymphocyte, and platelet values were available at the individual patient level in the study dataset.

### Clinical grouping

Three comparison groups were created to evaluate the association of the HALP score with pN and pT in different clinical contexts.

Group 1 compared patients with pN0 disease (n = 43) with those with pN+ disease (n = 57).

Group 2 compared patients with pT1–2/pN0 disease (n = 20) with all other patients (n = 80).

Group 3 compared patients with pT1–2 disease (n = 25) with those with pT3–4 disease (n = 75), irrespective of pN status.

### Statistical analysis

The distribution of continuous variables was assessed using the Shapiro–Wilk test. Continuous variables that were not normally distributed were presented as median (interquartile range), whereas categorical variables were presented as numbers and percentages. The pT1–2 and pT3–4 groups were compared using the Mann–Whitney U test for continuous variables and Fisher’s exact test for categorical variables. Hemoglobin, albumin, lymphocyte, platelet, and HALP values were evaluated separately between the two pT groups.

The discriminative performance of the HALP score was assessed using ROC analysis. AUC values were presented with 95% confidence intervals and p values; AUC confidence intervals were calculated using the DeLong method, and cut-off values were determined using the Youden index. Wilson-based 95% confidence intervals were used for sensitivity, specificity, positive and negative predictive values, and accuracy, whereas 95% confidence intervals for balanced accuracy were based on 20, 000 bootstrap resamples. A high HALP value was considered a positive test result for pN0 disease in Group 1, pT1–2/pN0 disease in Group 2, and pT1–2 disease in Group 3.

The HALP score was evaluated as a continuous variable per 10-unit increase, first in univariable analysis and then in multivariable logistic regression analysis adjusted for age, sex, and pN status. Age was entered into the model per 10-year increase. In addition, stratified logistic regression and ROC analyses were performed in patients with pN0 and pN+ disease.

Internal validation of the data-derived cut-off value was performed using 5, 000 bootstrap samples generated by sampling with replacement. In each resample, the optimal cut-off value was re-estimated using the Youden index and evaluated in out-of-bag observations. Median estimates and 2.5th–97.5th percentile intervals were reported. The multivariable model was refitted in 3, 000 bootstrap samples and evaluated in out-of-bag observations. A two-sided p value < 0.05 was considered statistically significant.

### Ethics committee approval

Ethics committee approval for the study was obtained from Ankara Bilkent City Hospital (Decision No: TABED 1-25-1028; Date: February 26, 2025). The study was conducted in accordance with the principles of the Declaration of Helsinki.

## Results

A total of 100 patients who underwent surgery for colon cancer between December 2020 and December 2025 were included in the study. Of the patients, 65 (65.0%) were male and 35 (35.0%) were female. The median age was 69.0 years (IQR: 62.0–74.25), with an age range of 27–89 years. According to pathological T category, 11 patients (11.0%) had pT1, 14 (14.0%) had pT2, 58 (58.0%) had pT3, and 17 (17.0%) had pT4 disease. Pathological lymph node evaluation revealed pN0 disease in 43 patients (43.0%) and pN+ disease in 57 patients (57.0%). According to the 8th edition of the AJCC TNM classification, 20 patients (20.0%) were classified as stage I, 23 (23.0%) as stage II, and 57 (57.0%) as stage III; no patients had stage IV disease [n = 0 (0.0%)]. All patients had M0 disease [n = 100 (100.0%)].

Regarding the surgical approach, open surgery was performed in 60 patients (60.0%), whereas laparoscopic surgery was performed in 40 patients (40.0%). The surgical procedures included right hemicolectomy in 39 patients (39.0%), left hemicolectomy in 20 (20.0%), sigmoid resection in 34 (34.0%), and transverse colectomy in 7 (7.0%). The median number of examined lymph nodes was 21.1 (IQR: 17.3–25.7). None of the patients had chronic inflammatory or hematological disease, preoperative transfusion, or hepatic/renal dysfunction, and none had received neoadjuvant treatment.

### ROC and cut-off analyses

**Figure 1:**
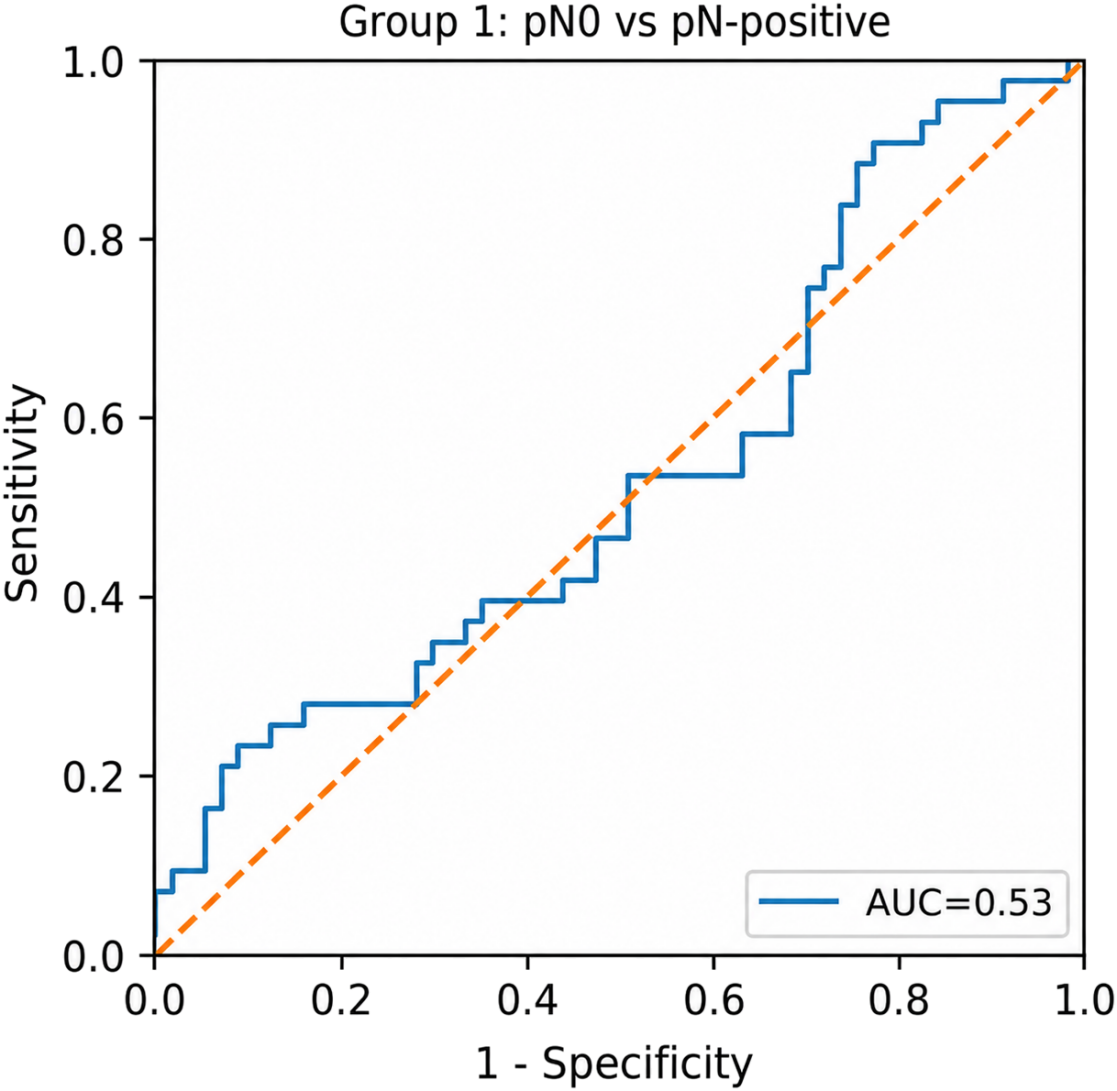
ROC curve of the HALP score in distinguishing patients with pN0 disease from those with pN+ disease.

**Figure 2:**
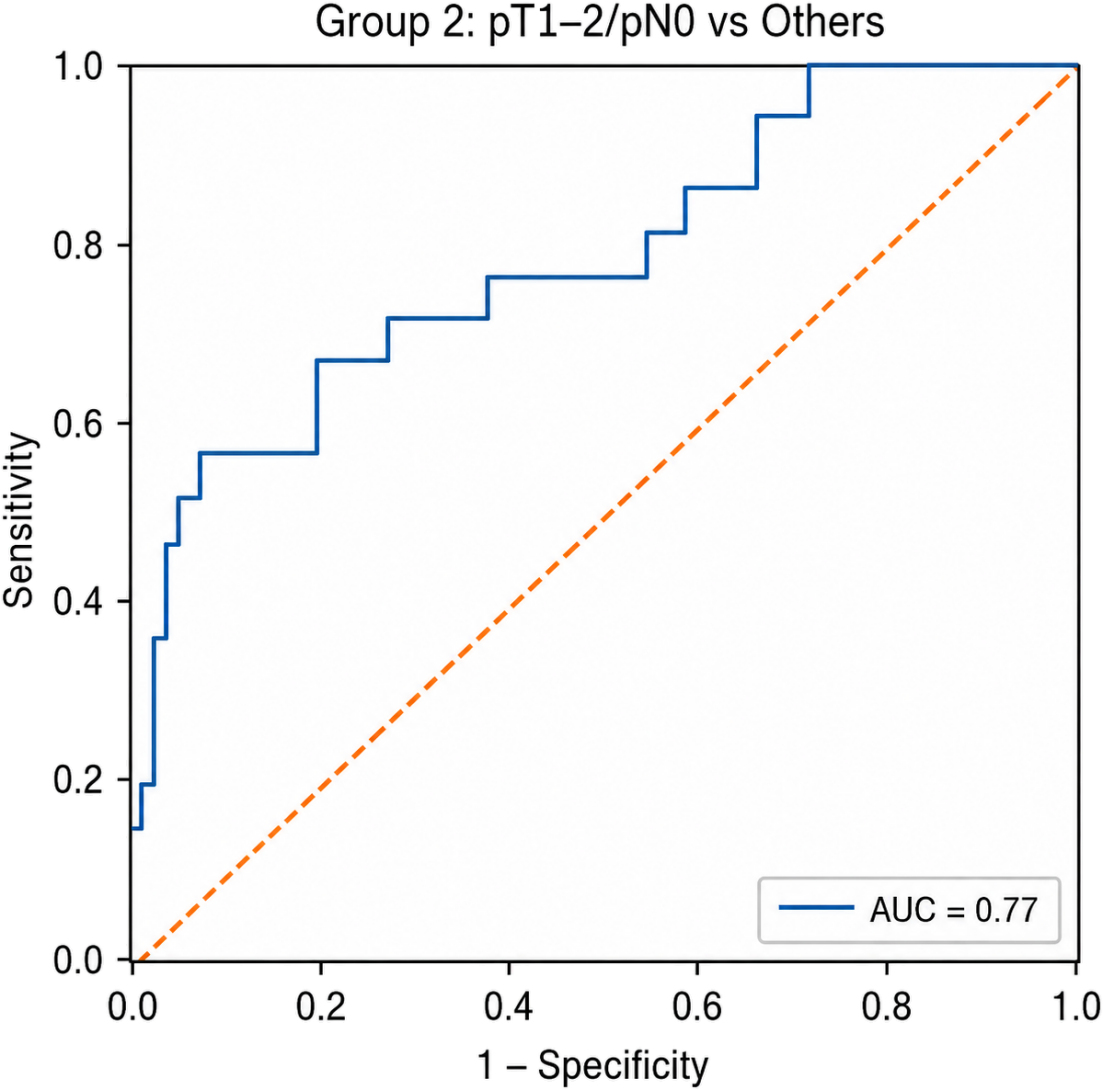
ROC curve of the HALP score in distinguishing patients with pT1–2/pN0 disease from all other patients.

**Figure 3:**
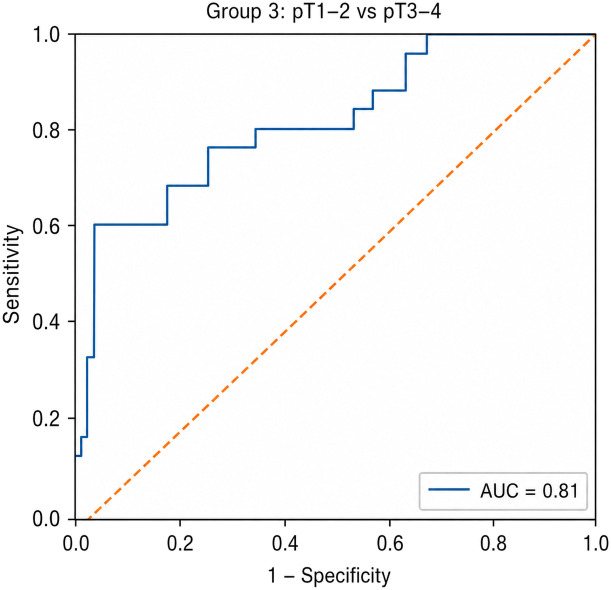
ROC curve of the HALP score in distinguishing patients with pT1–2 disease from those with pT3–4 disease.

The performance of the HALP score in distinguishing patients with pN0 disease from those with pN+ disease was not statistically significant in Group 1 (AUC = 0.53; 95% CI: 0.42–0.65; p = 0.568). In contrast, the AUC was 0.77 in Group 2, in which patients with pT1–2/pN0 disease were compared with all other patients (95% CI: 0.64–0.89; p < 0.001), and 0.81 in Group 3, in which patients with pT1–2 disease were compared with those with pT3–4 disease (95% CI: 0.71–0.92; p < 0.001). According to the Youden index, the optimal cut-off value was 59.38 for Group 1 and 55.37 for Groups 2 and 3 (**Table 1**). The corresponding ROC curves are shown in **Figures 1**–**3**.

**Table 1:** ROC performance of the HALP score in distinguishing pT and pN categories.

Comparison	Number of patients	Youden cut-off	AUC (95% CI)	P value
Group 1: pN0 vs pN+	43 vs 57	≥59.38	0.53 (0.42-0.65)	0.568
Group 2: pT1–2/pN0 vs others	20 vs 80	≥55.37	0.77 (0.64-0.89)	<0.001
Group 3: pT1–2 vs pT3–4	25 vs 75	≥55.37	0.81 (0.71-0.92)	<0.001

A high HALP value was defined as a positive test result for pN0 in Group 1, pT1–2/pN0 in Group 2, and pT1–2 in Group 3. AUC, area under the curve; CI, confidence interval.

### Diagnostic performance of the cut-off values

Although the overall accuracy of the data-derived cut-off values was 84.0% for Group 2 and 87.0% for Group 3, the corresponding sensitivity values were 55.0% and 60.0%, respectively. Balanced accuracy was 73.1% in Group 2 and 78.0% in Group 3. Because the ROC analysis was not statistically significant in Group 1, the cut-off-based classification metrics were not considered evidence of predictive performance (**Table 2**).

**Table 2:** Diagnostic performance of the HALP cut-off values identified in the study.

Group	Sensitivity, % (95% CI)	Specificity, % (95% CI)	PPV, % (95% CI)	NPV, % (95% CI)	Accuracy, % (95% CI)	Balanced accuracy, % (95% CI)
Group 1	23.3 (13.2-37.7)	91.2 (81.1-96.2)	66.7 (41.7-84.8)	61.2 (50.5-70.8)	62.0 (52.2-70.9)	57.2 (50.2-64.6)
Group 2	55.0 (34.2-74.2)	91.2 (83.0-95.7)	61.1 (38.6-79.7)	89.0 (80.4-94.1)	84.0 (75.6-89.9)	73.1 (61.2-84.6)
Group 3	60.0 (40.7-76.6)	96.0 (88.9-98.6)	83.3 (60.8-94.2)	87.8 (79.0-93.2)	87.0 (79.0-92.2)	78.0 (67.9-87.7)

PPV, positive predictive value; NPV, negative predictive value; CI, confidence interval.

### Internal validation

In the internal validation based on 5, 000 bootstrap resamples, the median cut-off value remained 55.37 for both Group 2 and Group 3. In Group 3, the percentile interval of the cut-off value was 30.16–59.38, which was narrower than the 18.93–64.38 interval observed in Group 2. In addition, the AUC of 0.81 (95% interval: 0.66–0.95), sensitivity of 58.0%, specificity of 87.4%, accuracy of 80.0%, and balanced accuracy of 72.7% in Group 3 were higher than those in Group 2. These findings demonstrated that the cut-off value of 55.37 showed more consistent and stable discriminative performance in Group 3 than in Group 2 (**Table 3**).

**Table 3:** Bootstrap validation of the HALP cut-off values identified in the study.

Comparison	Median cut-off (2.5th–97.5th percentiles)	AUC (95% CI)	Sensitivity, % (95% CI)	Specificity, % (95% CI)	Accuracy, % (95% CI)	Balanced accuracy, % (95% CI)
Group 2	55.37 (18.93–64.38)	0.77 (0.57–0.93)	52.3 (14.3–100.0)	81.6 (26.7–100.0)	75.9 (41.5–90.9)	67.0 (50.0–83.2)
Group 3	55.37 (30.16–59.38)	0.81 (0.66–0.95)	58.0 (25.0–90.0)	87.4 (56.2–100.0)	80.0 (60.6–93.3)	72.7 (58.1–87.0)

p, percentile.

The median age was 64.0 years (IQR: 61.0–69.0) in the pT1–2 group and 70.0 years (IQR: 63.0–75.0) in the pT3–4 group, indicating that patients in the advanced pT category were older (p = 0.020). Sex distribution did not differ significantly between the groups (p = 0.148). Similarly, the distribution of right, transverse, left, and sigmoid colon tumor locations was comparable between the pT1–2 and pT3–4 groups (p = 0.992). Pathological lymph node involvement was more frequent in the advanced pT group. The pN+ rate was 20.0% in the pT1–2 group and 69.3% in the pT3–4 group (p < 0.001). High histological grade was observed in 24.0% of the pT1–2 group and 30.7% of the pT3–4 group, with no statistically significant difference between the groups (p = 0.617). Although lymphovascular invasion positivity was also numerically higher in the pT3–4 group (25.3% vs 12.0%), this difference did not reach statistical significance (p = 0.264).

The median number of examined lymph nodes was 17.2 in the pT1–2 group and 22.3 in the pT3–4 group. Median hemoglobin, albumin, and lymphocyte levels were lower in the pathological T3–4 group, whereas platelet counts were higher. The median hemoglobin level was 12.4 g/dL (IQR: 11.2–13.7) in the pT1–2 group and 10.7 g/dL (IQR: 9.6–11.8) in the pT3–4 group. The corresponding median albumin levels were 32.8 g/L (IQR: 30.3–35.6) and 30.5 g/L (IQR: 28.1–33.1), median lymphocyte counts were 2.01 × 10^9^/L (IQR: 1.65–2.45) and 1.85 × 10^9^/L (IQR: 1.52–2.25), and median platelet counts were 149.1 × 10^9^/L (IQR: 128.7–179.4) and 224.9 × 10^9^/L (IQR: 190.5–265.5), respectively. None of the patients included in the study had a history of chronic inflammatory disease, hepatic or renal dysfunction, or preoperative transfusion. Comparative demographic, clinical, pathological, and laboratory characteristics of the pT1–2 and pT3–4 groups are presented (**Table 4**).

**Table 4:** Clinical and laboratory characteristics by pathological T category.

Variable	All patients (n=100)	pT1-2 (n=25)	pT3-4 (n=75)	P value
Age, years, median (IQR)	69.0 (62.0-74.25)	64.0 (61.0-69.0)	70.0 (63.0-75.0)	0.020
Sex				0.148
Male	65 (65.0)	13 (52.0)	52 (69.3)	
Female	35 (35.0)	12 (48.0)	23 (30.7)	
Tumor location, n (%)				0.992
Right colon	39 (39.0)	10 (40.0)	29 (38.7)	
Transverse colon	7 (7.0)	2 (8.0)	5 (6.7)	
Left colon	20 (20.0)	5 (20.0)	15 (20.0)	
Sigmoid colon	34 (34.0)	8 (32.0)	26 (34.6)	
Pathological T category, n (%)				
pT1	11 (11.0)	11 (44.0)	0 (0.0)	
pT2	14 (14.0)	14 (56.0)	0 (0.0)	
pT3	58 (58.0)	0 (0.0)	58 (77.3)	
pT4	17 (17.0)	0 (0.0)	17 (22.7)	
Pathological N category, n (%)				<0.001
pN0	43 (43.0)	20 (80.0)	23 (30.7)	
pN+	57 (57.0)	5 (20.0)	52 (69.3)	
Histological grade, n (%)				0.617
Low-grade (G1–G2)	71 (71.0)	19 (76.0)	52 (69.3)	
High-grade (G3–G4)	29 (29.0)	6 (24.0)	23 (30.7)	
Lymphovascular invasion, n (%)				0.264
Negative	78 (78.0)	22 (88.0)	56 (74.7)	
Positive	22 (22.0)	3 (12.0)	19 (25.3)	
Number of lymph nodes examined, median (IQR)	21.1 (17.3–25.7)	17.2 (14.1–21.0)	22.3 (18.3–27.2)	
Hemoglobin, g/dL, median (IQR)	11.1 (10.0–12.3)	12.4 (11.2–13.7)	10.7 (9.6–11.8)	
Albumin, g/L, median (IQR)	31.1 (28.7–33.7)	32.8 (30.3–35.6)	30.5 (28.1–33.1)	
Lymphocyte count, ×10^9^/L, median (IQR)	1.89 (1.55–2.30)	2.01 (1.65–2.45)	1.85 (1.52–2.25)	
Platelet count, ×10^9^/L, median (IQR)	206.6 (175.0–244.0)	149.1 (128.7–179.4)	224.9 (190.5–265.5)	
Chronic inflammatory disease	0 (0.0)	0 (0.0)	0 (0.0)	
Hepatic/renal dysfunction	0 (0.0)	0 (0.0)	0 (0.0)	
Preoperative transfusion	0 (0.0)	0 (0.0)	0 (0.0)	

The diagnostic performance of the 55.37 cut-off value for Group 3 was examined across age and sex subgroups. The specificity of the cut-off value was high in all subgroups, reaching 100.0% in women, 94.2% in men, 90.0% in patients younger than 65 years, and 98.2% in patients aged 65 years or older. Sensitivity was 50.0% in women and 69.2% in men, and 53.8% in patients younger than 65 years and 66.7% in those aged 65 years or older. No statistically significant differences were found between female and male patients in terms of sensitivity and specificity (p = 0.428 and p = 0.548, respectively). Similarly, sensitivity and specificity did not differ significantly between patients younger than 65 years and those aged 65 years or older (p = 0.688 and p = 0.172, respectively). No statistically significant differences in sensitivity or specificity were observed across the age and sex subgroups, and the specificity of the cut-off value was high in all subgroups. The sensitivity, specificity, positive predictive value, and negative predictive value of the 55.37 cut-off value for Group 3 across age and sex subgroups are presented in (**Table 5**).

**Table 5:** Diagnostic performance of the 55.37 HALP cut-off value for group 3 across age and sex subgroups.

Subgroup	Sensitivity	Specificity	PPV	NPV
Female (n=35)	50.0% (95% CI: 25.4–74.6)	100.0% (95% CI: 85.7–100.0)	100.0% (95% CI: 61.0–100.0)	79.3% (95% CI: 61.6–90.2)
Male (n=65)	69.2% (95% CI: 42.4–87.3)	94.2% (95% CI: 84.4–98.0)	75.0% (95% CI: 46.8–91.1)	92.5% (95% CI: 82.1–97.0)
Age <65 years (n=33)	53.8% (95% CI: 29.1–76.8)	90.0% (95% CI: 69.9–97.2)	77.8% (95% CI: 45.3–93.7)	75.0% (95% CI: 55.1–88.0)
Age ≥65 years (n=67)	66.7% (95% CI: 39.1–86.2)	98.2% (95% CI: 90.4–99.7)	88.9% (95% CI: 56.5–98.0)	93.1% (95% CI: 83.6–97.3)

PPV, positive predictive value; NPV, negative predictive value; CI, confidence interval.

In the univariable analysis, each 10-unit increase in the HALP score was associated with 2.08-fold higher odds of the pT1–2 category (OR = 2.08; 95% CI: 1.54–2.86; p < 0.001). This association remained significant in the model adjusted for age, sex, and pN status, and each 10-unit increase in the HALP score was associated with 3.23-fold higher odds of the pT1–2 category (adjusted OR = 3.23; 95% CI: 1.85–5.88; p < 0.001). In the adjusted analysis, age was not significant, whereas male sex and pN+ status were associated with lower odds of the pT1–2 category. In the nodal-stratified analyses, each 10-unit increase in the HALP score was associated with higher odds of the pT1–2 category in both patients with pN0 disease and those with pN+ disease. The performance of the HALP score in distinguishing between the pT1–2 and pT3–4 categories was AUC = 0.85 in patients with pN0 disease and AUC = 0.90 in those with pN+ disease. The AUC of the multivariable model was 0.94 in the original sample and 0.91 for out-of-bag observations across 3, 000 bootstrap resamples. The results of the regression analysis, nodal-stratified analyses, and internal validation are presented in (**Table 6**).

**Table 6:** Results of logistic regression, nodal-stratified analysis, and internal validation of the association between the HALP score and pathological T category.

Analysis	Variable	Univariable OR (95% CI)	p value	Adjusted OR (95% CI)	p value	AUC (95% CI)
Regression model	HALP score, per 10-unit increase	2.08 (1.54–2.86)	<0.001	3.23 (1.85–5.88)	<0.001	
	Age, per 10-year increase	0.75 (0.51–1.09)	0.127	0.84 (0.46–1.54)	0.563	
	Male sex (female as reference)	0.48 (0.19–1.20)	0.119	0.14 (0.03–0.64)	0.011	
	pN+ status (pN0 as reference)	0.11 (0.04–0.33)	<0.001	0.03 (0.004–0.23)	<0.001	
Nodal stratified analysis	pN0 (n=43; pT1–2/pT3–4: 20/23)	2.78 (1.43–5.26)	0.002			0.85 (0.74–0.97)
	pN+ (n=57; pT1–2/pT3–4: 5/52)	2.50 (1.30–4.76)	0.006			0.90 (0.78–1.00)
Internal validation of the model	Original sample performance					0.94
	Out-of-bag observations from 3, 000 bootstrap samples					0.91 (0.81–0.99)

The dependent variable was defined as the pT1–2 category (pT1–2 = 1; pT3–4 = 0). ORs for HALP and age were calculated per 10-unit and 10-year increases, respectively. The adjusted model included the HALP score, age, sex, and pN status. In the nodal-stratified analyses, the OR represents the association between each 10-unit increase in the HALP score and the pT1–2 category. Internal validation of the model was performed using 3, 000 bootstrap resamples. HALP, hemoglobin–albumin–lymphocyte–platelet; OR, odds ratio; CI, confidence interval; AUC, area under the curve; pT, pathological T category; pN, pathological lymph node status.

## Discussion

The principal finding of this study was that, although the preoperative HALP score showed poor performance in distinguishing patients with pN0 disease from those with pN+ disease, it was able to distinguish between early and advanced pT categories independently of lymph node status. In addition, the statistically significant association between the HALP score and pT category persisted after adjustment for age, sex, and pN status.

There is no fixed and universally accepted cut-off value for the HALP score in the literature. In a study evaluating disease-free survival in patients with colon cancer, this value was found to be 32.4 (5). In another study, a cut-off value of 15.0 was identified for overall survival in patients with colon cancer (6). This variation in the literature is considered to result from differences in patient population characteristics, tumor stage, treatment protocols, targeted clinical outcomes, and methods used to determine cut-off values. In the present study, to reduce heterogeneity within the patient population, patients with M0 colon cancer who had not received neoadjuvant treatment were evaluated, and the study objective was limited to pT and pN categories rather than multifactorial outcomes such as survival. This approach aimed to provide a clear and straightforward evaluation of the HALP score in a homogeneous patient population using specific pathological outcomes. Another reason for the differences in HALP cut-off values reported across studies is the methods used for their calculation. In some studies, cut-off values were determined using the median value, whereas ROC analysis and the Youden index were used in others. In our study, a cut-off value of 55.37 was identified by ROC analysis for distinguishing between early and advanced pT categories independently of lymph node status. However, because this cut-off value was both derived and evaluated in the same patient population, it carries a risk of overfitting. Therefore, the value of 55.37 should be regarded not as a definitive or universal clinical cut-off, but as an exploratory finding specific to the current study cohort.

The HALP score is a biomarker that reflects the combined systemic inflammatory, immunological, and nutritional effects of the disease rather than the anatomical location of the tumor. Therefore, the effect of tumors located in different colonic segments on the HALP score was considered likely to be limited. However, the current literature lacks sufficient data in which the HALP score has been evaluated separately according to segmental colonic location, and no standardized segment-based analytical approach has been recommended. The inclusion of all colonic tumor locations within a single group in our study should be interpreted within this context. Nevertheless, the potential effect of different tumor locations within the colon on the HALP score should be further investigated in studies with larger sample sizes. Neoadjuvant treatment may lead to reductions in hemoglobin, lymphocyte, and platelet levels as a result of chemotherapy- and/or radiotherapy-induced bone marrow suppression and may affect albumin levels through loss of appetite, inadequate nutrition, and changes in systemic inflammation (7). However, in some studies in the literature, the neoadjuvant treatment status of patients has not been reported in sufficient detail. This issue is particularly important in colorectal cancer series because neoadjuvant treatment is widely used in rectal cancer but has a more limited role in colon cancer (8). Our study included only patients with colon cancer, and none of the patients in the cohort received neoadjuvant treatment. Thus, the potential confounding effect of neoadjuvant treatment on the components of the HALP score was reduced.

In a study of patients with colon cancer, a low HALP score was reported to be associated with advanced stage and to potentially reflect the anatomical extent of the disease (9). However, pTNM stage grouping combines the depth of tumor invasion into the bowel wall and regional lymph node metastasis within the same classification system. Although these two components are related, they do not represent the same biological characteristic of the tumor. Progression of tumor invasion may directly affect the components of the HALP score through increased local tumor burden, tissue damage, systemic inflammation, and nutritional deterioration. In contrast, lymph node metastasis depends not only on the depth of local invasion but also on the tumor’s capacity for lymphatic spread and its biological characteristics. Therefore, an association identified between the HALP score and overall pTNM stage does not clarify whether this relationship is driven by the T or N component, and a general staging interpretation based on the HALP score may be misleading. The occurrence of lymph node metastasis in early pT categories also indicates that pT and pN categories do not represent the same biological process. In the literature, lymph node metastasis has been reported in approximately 10% of T1 colon cancers and at rates reaching 25.8% in T2 tumors (10, 11). In our study, lymph node metastasis was detected in 2/11 (18.18%) patients with T1 disease and in 3/14 (21.42%) patients with T2 disease. These findings support that an early pT category does not always indicate lymph node-negative disease. To demonstrate this distinction, Groups 2 and 3 were established separately in our study. In Group 2, patients with pT1–2/pN0 disease were compared with all other patients to evaluate early-stage disease. In Group 3, early and advanced pT categories were compared irrespective of pN status. The inability of the HALP score to distinguish patients with pN0 disease from those with pN+ disease in Group 1, while showing better discrimination between pT1–2 and pT3–4 patients in Group 3, suggested that the association between the HALP score and the anatomical extent of the disease may primarily be attributable to the depth of local tumor invasion rather than lymph node status. In addition, the numerically higher discriminative performance of Group 3 compared with Group 2 supported that adding the pN0 condition to the early T category did not improve the performance of the HALP score. In the multivariable regression analysis, the association between the HALP score and the early pathological T category remained independent of age, sex, and pN status. The finding that each 10-unit increase in the HALP score was associated with higher odds of the pT1–2 category supported that higher HALP values may reflect more limited local tumor invasion. The persistence of this association in the same direction in separate analyses of patients with pN0 and pN+ disease, together with the high discriminative performance observed in both nodal strata, indicated that the association between the HALP score and pathological T category was not solely attributable to lymph node status. However, the small number of patients with pT1–2 disease in the pN+ group requires cautious interpretation of the results from this subgroup. The high AUC of the multivariable model in the original sample and the largely preserved performance after bootstrap internal validation supported the consistency of the model within the current cohort. Nevertheless, because pathological pN status was included in the model, this performance should not be interpreted as evidence of the success of a direct preoperative prediction model, but rather as a finding demonstrating the robustness of the adjusted association between the HALP score and pathological T category.

The association between the HALP score and pT category may be explained by the depth of local invasion represented by the pT category and by the systemic inflammatory and nutritional effects induced by the tumor, rather than directly by tumor diameter. Tumor diameter was not examined in our study; therefore, the direct association between the HALP score and tumor size was not evaluated. The hematological and biochemical parameters that constitute the HALP score are known to be influenced by age and sex. Hemoglobin levels have been reported to be lower in women, albumin levels lower at advanced age, and platelet counts higher in women (12–14). A study evaluating the HALP score reported that cut-off values may be influenced by demographic factors such as age and sex and may contribute to heterogeneity across studies (15). In our study, the cut-off value of 55.37 showed similar diagnostic performance across age and sex subgroups, with no statistically significant differences between the groups. This finding supported the objective and consistent performance of the cut-off value identified in the study across different demographic groups. The HALP score is calculated using a proportional mathematical formula based on hematological and nutritional parameters. Therefore, the absence of a marked difference between age and sex subgroups may be expected. Nevertheless, because this was a single-center study with a limited sample size, the performance of the cut-off value, particularly in female patients and those aged over 65 years, should be validated in larger cohorts.

The HALP score is an immunonutritional biomarker that can be easily calculated from routine preoperative blood tests, including hemoglobin, albumin, lymphocyte, and platelet values, without requiring additional cost or specialized testing. Owing to these characteristics, it can be readily integrated into daily clinical practice and may allow the patient’s immunological and nutritional status to be assessed using a single value. The association identified between the HALP score and pathological T category in our study suggests that this score may have potential as a practical biomarker to assist preoperative risk stratification in addition to current clinical and radiological assessments. However, because the HALP score was not directly compared with radiological and clinical staging or with other inflammatory and nutritional indicators, its incremental contribution beyond these methods was not evaluated in the present study. Nevertheless, the HALP score should not be regarded as a stand-alone staging or clinical decision-making tool, but rather as an adjunctive parameter that should be interpreted together with other clinical and radiological findings.

This study has several limitations. First, the retrospective, single-center design and the limited sample size may restrict the generalizability of the findings. Although the cut-off value of 55.37 identified in this study was internally validated using the bootstrap method, external validation in an independent patient cohort is required because it was derived from the same cohort. The small number of patients and wide confidence intervals in the age- and sex-based subgroup analyses require these results to be interpreted as exploratory. In addition, the lack of tumor diameter assessment and the absence of direct comparisons between the HALP score and radiological staging methods or other inflammatory and nutritional biomarkers limit the ability to determine the incremental clinical value of the score.

## Conclusion

In patients with colon cancer, the preoperative HALP score did not significantly distinguish patients with pN0 disease from those with pN+ disease. In contrast, an association between the HALP score and pT category was identified independently of age, sex, and pN status, and the score demonstrated significant performance in distinguishing patients with pT1–2 disease from those with pT3–4 disease. In our study, a cut-off value of 55.37 was identified for distinguishing between the pT1–2 and pT3–4 categories, and this value showed high specificity. The HALP score, which can be easily calculated from routine preoperative blood parameters, has the potential to serve as a practical immunonutritional biomarker reflecting the depth of local tumor invasion and supporting preoperative risk assessment in colon cancer. These findings suggest that the HALP score may be an exploratory biomarker associated with pT category and should be validated in larger, prospective, multicenter cohorts.

## Data Availability

The raw data supporting the conclusions of this article will be made available by the authors, without undue reservation.
